# Is the Pediatric Eosinophilic Esophagitis Symptom Score v2.0 reliable for telemedicine?

**DOI:** 10.55730/1300-0144.5649

**Published:** 2023-05-25

**Authors:** Sinem POLAT TERECE, Dilek YAPAR, Hacer İlbilge ERTOY KARAGÖL, Gizem KÖKEN, Demet TEKER DÜZTAŞ, Ödül EĞRİTAŞ GÜRKAN, Sinan SARI, Buket DALGIÇ, Arzu BAKIRTAŞ

**Affiliations:** 1 Division of Pediatric Allergy, Department of Pediatrics, Faculty of Medicine, Gazi University, Ankara, Turkiye; 2 Muratpaşa District Health Directorate, Antalya, Turkiye; 3 Division of Pediatric Gastroenterology, Department of Pediatrics, Faculty of Medicine, Gazi University, Ankara, Turkiye

**Keywords:** Children, eosinophilic esophagitis, reliability, telemedicine

## Abstract

**Background/aim:**

Eosinophilic esophagitis (EoE) is a chronic immune-mediated disease. Telemedicine is a healthcare technology used when a patient is separated by distance. The reliability of the Pediatric Eosinophilic Esophagitis Symptom Score, version 2.0 (PEESS v2.0) for telemedicine applications, has not been studied yet. Therefore, we aimed to evaluate the reliability of PEESS v2.0 for telemedicine.

**Materials and methods:**

We sent a telesurvey using questionnaires via electronic telecommunication as the telemedicine method. Children with EoE and their parents were asked to complete PEESS v2.0 with the telesurvey method (unsynchronized with the physician) and attend in-person visits one week apart. Intraclass correlation (ICC), Wilcoxon, and Bland–Altman tests were used as reliability analyses. Reliability was defined as a strong agreement between the measurements in ICC ≥ 0.8 and a p-value of ≤0.05 and no statistically significant difference between the scores of the two methods in the Wilcoxon and Bland–Altman analyses, i.e. a p-value of >0.05.

**Results:**

The total scores of children and parents were higher in in-person visits than in the telesurvey (Wilcoxon tests, p ≤ 0.05). Bland–Altman analysis showed that the mean difference in total scores between the two methods was significant for both children and parents (p ≤ 0.05). ICC levels for the children and parent scores for the entire group ranged from 0.595 to 0.763 (moderate agreement).

**Conclusion:**

Unsynchronized telesurvey use of PEESS v2.0 is unreliable both for children and parents. We suggest testing the reliability of chosen telemedicine methods before using them in clinical and research practice.

## 1. Introduction

Eosinophilic esophagitis (EoE) is a chronic immune-mediated esophageal disease characterized clinically by symptoms related to esophageal dysfunction and histologically by an eosinophil-predominant inflammation [1]. EoE is common, and incidence ranges from 1 to 20 cases per 100.000 persons in the pediatric population [2–5]. Symptoms of EoE may vary depending on age; infants may display failure to thrive, feeding difficulties, nausea, vomiting, and abdominal pain. In older children, heartburn, chest pain, and early manifestations of dysphagia (such as slow and picky eating) are expected. In adolescents, symptoms become specific to esophageal narrowing, with solid food dysphagia and food impaction, similar to what is seen in adults [6].

Standardized and validated instruments that measure patient-reported outcomes (PROs) are needed for clinical use and research purposes. PROs are reported by the patient and/or a parent proxy for children.

Two instruments are recommended for evaluating symptoms in children and adolescents with EoE and assessing EoE-specific quality of life: the “Pediatric Eosinophilic Esophagitis Symptom Score, version 2.0 (PEESS v2.0)” and the Pediatric Quality of Life Inventory (PEDsQL) EoE module in “Core Outcomes Set for Therapeutic Studies in Eosinophilic Esophagitis (COREOS)” [7]. GaziESAS is another parent-proxy instrument that was developed to evaluate adaptive behavior for coping with dysphagia in addition to assessing symptom frequency in children with EoE; the scale’s improved validity and reliability have been tested on 84 patients [8]. These instruments are useful in telemedicine, especially in situations such as the pandemic, where patients have limited access to hospitals, as well as in the case of out-of-town patients who need remote clinical follow-ups.

Telemedicine is defined as the use of technology to deliver health care, health information, or health education to a patient who is separated by distance or time [9]. Telemedicine technologies have been proven to work and are considered a viable option for healthcare delivery [10]. Due to the restrictions brought about by the coronavirus disease 2019 (COVID-19) pandemic, it has become more common to provide health monitoring with telemedicine in children with chronic diseases [11]. The prolonged quarantine period during the COVID-19 pandemic has hindered the ability of our EoE patients to come to clinics. This restriction and challenges have necessitated the use of telemedicine methods for our patients. Telesurveys, in which questionnaires are used via electronic telecommunication, is one of the applications of telemedicine [12,13]. Although telesurveys have already begun to be used in allergy practice, few studies have been performed on children with EoE [14–16]. However, the reliability of an EoE-specific symptom assessment via telesurvey methods has not yet been evaluated. Therefore, there arose a need to test the reliability of the most frequently used symptom scale (PEESS v2.0) we will be using for telemedicine.

In this study, we aimed to test the reliability of the telesurvey administration of the Turkish version of PEESS v2.0 (Tr-PEESS v2.0) to both families and children.

## 2. Materials and methods

### 2.1. Definitions

The diagnosis of EoE is based on symptoms of esophageal dysfunction and at least 15 eosinophils per high-power field (hpf) on esophageal biopsy, excluding non-EoE disorders that could cause or potentially contribute to esophageal eosinophilia [17]. In the absence of symptoms related to EoE, patients are considered symptomatically controlled. Histopathologic control (remission) is defined as the eosinophil count returning below 5 eosinophils per hpf in all biopsies from the upper, middle, and lower esophagus.

### 2.2. Instrument

The PEESS v2.0 is a scale that evaluates the frequency and severity of symptoms in the last month in children with EoE. There are two forms of PEESS v2.0: a single children/teens form (age 8–18 years) and a parent form (for children aged 2–18 years). The PEESS v2.0 is composed of 20 items investigating the two domains of EoE symptom frequency and severity. The domain scores range from 0 to 100. As the frequency and severity of EoE symptoms increase, the symptom score increases. Our group demonstrated the validity and reliability of PEESS v2.0 in Turkish [8]. In this study, the Turkish version of PEESS v2.0 was administered to all participants, permission for which was provided by MAPI Research Trust, Lyon, France (https://eprovide.mapi-trust.org).

### 2.3. Design of the study and participants

This methodologic study was designed prospectively by the Gazi University Pediatric Eosinophilic Gastrointestinal Diseases Working Group in March 2022. The sample size of the study was calculated using the G*Power 3.0.10 program. Assuming the estimated correlation coefficient as r = 0.6 (effect size) α error = 0.05 and power 0.8 (1-β error), the minimum sample size to be reached was calculated as 17 for each group (min. 34 in total). The sample was selected from March through April 2022 from children with EoE, who were scheduled for a clinical follow-up. Tr-PEESS v2.0 has been used at least three times at our center on the same group of parents and patients for different studies; therefore, the participants were familiar with the scale. In our previous PEESS v2.0 study, we realized that adolescents aged ≥12 years had better reading/writing and comprehension skills than the younger group (elementary and middle school group) [8]. For this reason, in the present study, we evaluated the reliability of PEESS v2.0 through a telesurvey in children (age <12 years) and teens (≥12 years) separately, in addition to parents. Children diagnosed with EoE, aged ≥8 years, and their parents/guardians who had access to a computer or phone with email capabilities, and gave informed consent were included in the study. Children with EoE who were under the age of 8 were excluded from the study due to the lack of a scale applicable to this age group. Additionally, individuals with cognitive and neurodevelopmental impairments alongside EoE, those taking medications that could affect their cognitive functions, individuals who did not provide consent to participate in the study, and those who did not have access to a computer or phone with email capabilities were also excluded from the study.

### 2.4. Survey administration

PEESS v2.0 forms for children and parents were sent to the families by email one week before the scheduled follow-up visit. They were asked to complete the scale and return it within a day. The same forms were completed again during in-person visits by the participants. Informed consent was obtained. There were no differences in the clinical conditions or treatments of the patients during the study period.

### 2.5. Statistical analysis

Statistical analysis was performed using the SPSS v23.0 software package (IBM Corp., Armonk, NY, USA). Descriptive statistics are presented as numbers and percentages for categorical variables and median (minimum value – maximum value) for continuous variables. A p-value of ≤0.05 was considered statistically significant. The reliability analyses of PEESS v2.0 via telesurveys was evaluated using three different statistical methods: intraclass correlation (ICC), the Wilcoxon test, and the Bland–Altman test. In this study, the telesurvey method needed to pass all three tests to be considered a reliable method. Generally, ICC, which is a reliability index, measures the agreement between methods (tested method vs. gold standard method) using the 2-way fixed effects model (absolute agreement). A p-value of >0.05 indicates a lack of agreement between two methods and p ≤ 0.05 is interpreted to indicate agreement. ICC values less than 0.5 were considered to have poor reliability, values between 0.5 and 0.8 were considered to show moderate reliability, and values above 0.8 and 0.9 were regarded as a sign of good or excellent reliability [18,19]. For a measure or method to be considered reliable, there must be strong agreement (ICC ≥0.8) between the measurements. In the Wilcoxon and Bland–Altman tests, a p-value of ≤0.05 indicates that the method is not reliable. In addition to its use as a reliability test, the Wilcoxon test also assesses the validity of the telesurvey method, and a statistically significant difference between the scores obtained via the telesurveys and in-person visits shows that the tested method is not valid.

## 3. Results

A total of 36 participants (10 males / 8 females with EoE and their parents) were included in the study. The median age of the patients was 140 (range, 102–215) months, and half of the patients (n = 9) were aged <12 years. The median follow-up was 51.4 (range: 11–146) months. Eight (44.4%) patients had allergic comorbidities (asthma: n = 5; allergic rhinitis: n = 5; atopic dermatitis: n = 1). A family history of EoE was present in three patients (16.6%). Nine (50%) patients were under treatment (proton pump inhibitors: n = 6; topical swallowed budesonide: n = 1; diet: n = 2). Nine patients refused to have any treatment. The histopathologic control (remission) rate was 44.4% (n = 8). There was a strong correlation between parents and patients in both in-person (r = 0.82, p < 0.001) and telemedicine applications (r = 0.81, p < 0.001), respectively.

**Figure 1:**
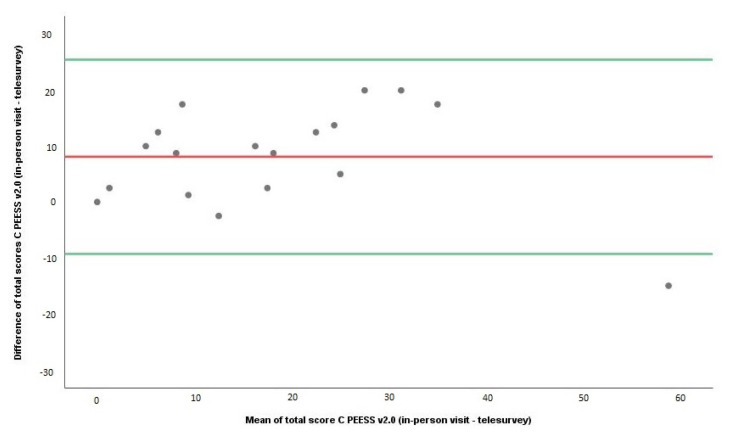
Bland–Altman plots of PEESS v2.0 scores of children (8–18 years)

**Figure 2:**
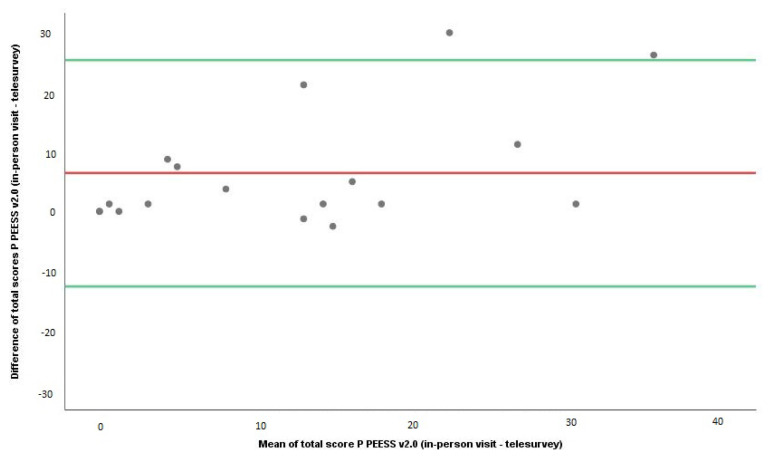
Bland–Altman plots of PEESS v2.0 scores of parents.

The reliability analysis of PEESS v2.0 by the telesurvey method is given in Table, Figure 1, and Figure 2. The first test for reliability analysis was ICC. It was noted that the ICC levels for the child and parent scores for the entire group, regardless of age, ranged from 0.595 to 0.763 (moderate agreement) (Table). The second test for reliability analysis was the Wilcoxon test. The total scores for both children and parents in the in-person application of PEESS v2.0 were statistically and significantly higher than those on the telesurvey (Wilcoxon tests, p ≤ 0.05) (Table). The third test for reliability analysis was the Bland–Altman test. The results of the Bland–Altman test and plots showed that the mean difference in total PEESS v2.0 scores between in-person visits and the telesurvey method was statistically significant for both children and parents (Table) (Figures 1 and 2).

## 4. Discussion

This is the first study to evaluate whether the use of PEESS v2.0 in telemedicine is reliable. Total PEESS v2.0 scores were significantly different between telesurveys and in-person visits and in moderate agreement both in parents and children. Therefore, we found the telesurvey use of both children/teen and parent forms of PEESS v2.0 unreliable. The reliability of assessment tools and techniques affects the outcomes of clinical trials and research studies [20,21]. Although telesurvey applications have been used in EoE in recent years [16,22–24], their reliability has not been tested. This is the first study to evaluate the reliability of the application of PEESS v2.0 in telesurveys. Total PEESS v2.0 scores between telesurveys and in-person visits were significantly different in both parents and children. The agreement between the two applications was only moderate.

In this study, we think that the lack of reliability of the telesurvey use of the scale may be explained in two ways. The first of these is the environment in which the instrument was administered. A healthcare institution may reinforce a greater perception and ability to scrutinize the condition of health by patients and their caregivers in contrast to many distracting stimuli at home or work environment. The second reason may be related to the telesurvey use of PEESS v2.0 in an unsynchronized manner. The synchronous application of PEESS v2.0 in telesurveys in real-time by a telephone call or video conference may have changed the results. A synchronized telesurvey application may be effective in ensuring the time that the questionnaire is completed within the allocated period, in reducing outside stimuli, and in increasing the participant’s concentration and cooperation [25]. In addition, synchronized telesurvey applications result in greater patient satisfaction than unsynchronized applications [25].

Although PEESS v2.0 by telesurvey was not found to be reliable for children aged 8–18 years for whom the original scale developed, in subgroup analysis, it was shown to be reliable in an older age group (>12 years old). We believe that the short span of concentration and weaker cognitive functions of children aged under 12 years might lead to the unreliability of the scale by telesurvey both in this young age group and all child age groups [26]. Unfortunately, we can not use the same explanation for the parents’ results.

There is only one other study that used PEESS v2.0 via a telemedicine method. In the study, PEESS v2.0 was sent to 90 patients with EoE every month through a message linked to a questionnaire that could be completed online in an unsynchronized manner. The study aimed to compare the management outcomes of EoE in these patients (n = 90; PEESS group) with those who were not sent PEESS v2.0 (n = 530, non-PEESS group) [16]. The authors found a statistically significant difference between the groups in favor of the PEESS v2.0-applied group compared with the non-PEESS v2.0 group in terms of the number of pediatric gastroenterology presentations and endoscopy procedures. The feasibility of a telemedicine method, including usability from a patient perspective, and reliability compared with in-person visit administration is a mandatory step before it can be recommended for clinical and research purposes [27]. However, no study involving unsynchronized telemedicine use of PEESS v2.0 has evaluated whether it is reliable enough to be used instead of in-person visits before using it [16].

The strongest aspect of our study is that the reliability of the telesurvey method was tested using three different reliability-testing methods (Bland-Altman test, ICC, and the Wilcoxon test) [28]. The ICC is supported by variance analysis. Therefore, in populations yielding a high ICC value, good correlation levels may not point to a good agreement. In reliability studies using the Bland–Altman or Wilcoxon tests, results are not affected by variance in the population and are more subjective. This is why combining reliability testing methods produces more accurate results [29]. High power (91%) and effect size (0.83) is also strength of this study. Also, the studied disease group, EoE is included under rare diseases spectrum. Furthermore, the lack of studies on the reliability of applying any EoE scale using telemedicine methods to both pediatric and adult cases of EoE. is another strong aspect. Perhaps the most significant contribution of this study to the literature is being the first study to investigate the reliability of using the most commonly used symptom scale (PEESS v2.0) in EoE through a telemedicine method.

The major limitation of the study was the lack of synchronized use of PEESS v2.0 alongside an unsynchronized arm as another telesurvey method. We think that the unreliability of an unsynchronized application may not be unique to PEESS v2.0; however, we could not provide data supporting this hypothesis. Although the sample size has been planned to be sufficient and analytically powerful enough to answer the research questions and meet the objectives of the study, we acknowledge that using a larger sample could enhance the statistical power and generalizability of our findings.

In conclusion, although PEESS v2.0 is reliable in terms of follow-up of symptoms in in-person applications, an unsynchronized telesurvey is not a reliable instrument for children or parents. Therefore, we suggest testing the reliability of chosen telemedicine methods before using them in clinical practice and research areas.

## Figures and Tables

**Table:** Reliability analysis of PEESS v2.0 by telesurvey.

PEESS v2.0 total scores	ICC (95% CI) p	Wilcoxon test Telesurvey In person p	Bland–Altman Mean difference (In person Telesurvey) p
**Parent**	0.595 (0.14–0.83) **0.001**	6.9 (0–30) 13.1 (0–48.8) **0.005**	6.4 ± 9.7 **0.012**
**Children**	0.727 (0.16–0.90) **<0.001**	13.8 (0–66.3) 20.0 (0–51.3) **0.004**	8.1 ± 8.9 **0.001**
**<12 years**	0.691 (0.08–0.93) **0.001**	13.8 (0–26.3) 21.3 (0–43.8) **0.012**	9.3 ± 6.8 **0.003**
≥**12 years**	0.763 (0.26–0.94) **0.003**	13.8 (0–66.3) 17.5 (10–51.3) 0.110	6.8 ± 11.1 0.101
